# Suicidality Related to the COVID-19 Lockdown in Romania: Structural Equation Modeling

**DOI:** 10.3389/fpsyt.2022.818712

**Published:** 2022-05-17

**Authors:** Anca-Livia Panfil, Diana Lungeanu, Simona Tamasan, Cristina Bredicean, Ion Papava, Daria Smirnova, Konstantinos N. Fountoulakis

**Affiliations:** ^1^Liaison Psychiatry, “Pius Brinzeu” County Emergency Hospital, Timisoara, Romania; ^2^Department of Functional Sciences, Center for Modeling Biological Systems and Data Analysis, “Victor Babes” University of Medicine and Pharmacy, Timisoara, Romania; ^3^Discipline of Psychiatry, Department of Neuroscience, NEUROPSY-COG Center for Cognitive Research in Neuropsychiatric Pathology, “Victor Babes” University of Medicine and Pharmacy, Timisoara, Romania; ^4^Psychiatry Compartment, “Dr. Victor Popescu” Emergency Military Clinical Hospital, Timisoara, Romania; ^5^“Eduard Pamfil” Psychiatry Clinic, “Pius Brinzeu” County Emergency Hospital, Timisoara, Romania; ^6^International Centre for Education and Research in Neuropsychiatry (ICERN), Samara State Medical University, Samara, Russia; ^7^Department of Psychiatry, Narcology, Psychotherapy and Clinical Psychology, Samara State Medical University, Samara, Russia; ^8^3rd Department of Psychiatry, School of Medicine, Aristotle University of Thessaloniki, Thessaloniki, Greece; ^9^Mental Health Section, Research Institute, Panhellenic Medical Association, Thessaloniki, Greece

**Keywords:** suicide, suicidal ideation, SEM, anxiety, depression, self-harm behavior

## Abstract

**Background:**

Suicidality is a serious public health concern at a global scale. Suicide itself is considered to be preventable death; worldwide, suicide rates and their trends are under constant scrutiny. As part of the international COMET-G cross-sectional study, we conducted a national level investigation to examine the individual disturbances (such as anxiety, depression, or history of life-threatening attempts) and contextual factors (such as adherence to conspiracy theories or Internet use) associated with suicidality related to the COVID-19 lockdown in a lot of Romanian adults.

**Participants and Methods:**

One thousand four hundred and forty-six adults responded to an anonymous on-line questionnaire, with mean age ± standard deviation of 47.03 ± 14.21 years (1,142 females, 292 males, 12 identified themselves as non-binary). Data were analyzed using descriptive statistics and structural equation modeling (SEM).

**Results:**

Univariate analysis showed strong significant correlation between anxiety and depression scorings among the respondents (Spearman R = 0.776, *p* < 0.001). Both the suicidality scorings and the Internet use correlated fairly with anxiety and depression, with two-by-two Spearman coefficients between R = 0.334 and R = 0.370 (*p* < 0.001 for each). SEM analysis substantiated the emotional disturbances, previous life-threatening attempts, and younger age as significant predictors for suicidality. The patterns of reality reading (including religious inquiries, Internet use, and beliefs in conspiracy theories) did not reach the statistical significance as influential factors in the suicidality of these respondents. There was no covariance between the Internet use and belief in conspiracy theories.

**Conclusion:**

The study confirmed the suicidality risk initially hypothesized as being associated with the history of life-threatening attempts, increased depression within the younger population, and higher anxiety during the first year of the COVID-19 pandemic and its related lockdown. National strategies for effective interventions at various levels of the healthcare system should be developed.

## Introduction

Suicidality is a serious public health concern at the global scale, affecting millions of people, their families, and society itself (1). The term “suicidality” includes suicidal ideation (SI, such as serious thoughts about taking one's own life), suicide plans, and suicide attempts (2). Significant resources and efforts have been focused on a better understanding of its underlying etiology, assessing the risks, and designing effective solutions at different levels of interventions (3). Suicide itself has been linked to the well-documented psychopathological risk predictors (such as suicidal behavior, history of self-harm and suicidal attempts), but there also are wide variations in suicidality indicators and suicide rates across countries and cultural environments (2, 4–13). Compared to other parts of the world, Europe is characterized by relatively high suicide rates, namely 10.5 (8.3–13.6) per 100,000 people per year (11, 12, 14). Depression has been acknowledged as a major risk factor for suicidality (15) and several studies have pointed toward anxiety as a major risk factor as well (16, 17). Demographic factors (e.g., young age, male gender, or ethnicity), social status (e.g., low income, income inequality, unemployment, low education, and low social support), social changes, neighborhood (e.g., inadequate housing, overcrowding, or violence), and adverse environmental events (e.g., climate change, natural catastrophe, war, conflict, and migration) were also linked to suicidality. Surprisingly, reported global trends for suicide rates and suicidal behavior demonstrated a stability not only before, but also in the early months of the COVID-19 pandemic (18).

On the other hand, the COVID-19 pandemic onset had a disruptive impact on societies, with global devastating consequences (19). As more and more countries instituted total lockdown, various reports pointed out that such measures exacerbated mental health issues, although the interventions were acknowledged as necessary and effective in stopping the spread of the virus (20–22). Some researchers focused on segments of the population at a higher risk, such as the youth or the frontline healthcare workers (23–26). Furthermore, the general population experienced exacerbated anxiety, with additional symptoms of depression, psychosis, panic attacks, trauma and suicidal ideation that seemed to exceed the experience in the previous SARS and MERS outbreaks (27). There were case reports of unusual neuropsychiatric manifestations like catatonia (28), but results regarding the rates of suicide behavior, attempts, ideation, and self-harm during the COVID-19 pandemic have varied and have been inconclusive (18, 29). The dramatic societal changes, serious environmental incidents, and a rise in family violence have been registered among the most influential factors highly correlated with the suicide risks during the period and after the COVID-19 total lockdown (30–32). Consequently, there were several warnings issued regarding the mental health in general (33) and suicidality in particular (34). In extraordinary times, such as the COVID-19 pandemic and the associated lockdown, suicide was rather unpredictable, with questionable dynamic rates. The suitable timeframe for assessing causal psychological changes and factors' inter-relationships arouse controversy over the gauging limitations, although memory-based retrospective assessment on behavioral and complex emotionality would offer the means to circumvent the distorting irrelevant momentary details and grant a respite for the emotions to settle and restructure (35).

### Suicidality in Romania

In 2019, Romania reported an age-standardized suicide rate of 7.3 per 100,000 people per year, thus falling under the global age-standardized suicide rate of 10.5 per 100,000 people per year (14). Reported trends for suicide rates had been constantly decreasing since 2012, though with a consistent difference between sexes (i.e., females had a much lower rate than men) (36). To our knowledge, data on suicide risk factors in Romanian adult population has been scarce and of suboptimal quality.

On 16 March 2020, a state of national emergency was declared in Romania and total lockdown was instituted for 60 days, which brought a considerable burden of mental health consequences. A large community of migrant workforce in the Western Europe (over three million citizens), who massively returned home when the pandemic began, made Romania unique among the countries in European Union. Additional hurdles challenged the implementation of the protective measures: intrinsic weaknesses of the national healthcare system (e.g., aging infrastructure, low national health expenditure, and reported corruption), and one of the most religious populations in Europe (37, 38). Most Romanians identify themselves as Orthodox Christian, a highly conservative denomination, which was slow to react during this crisis (39). Notwithstanding these characteristics, psychological investigations in this period have reported the general population as being stable (40, 41), although actual information on suicide and suicidality is still too little.

### Objective of the Study

In this paper we report the results of a national sub-set analysis comprised in the international COMET-G study (*COVID-19 MEntal health inTernational for the General population*) and based on the data from the Romanian population. In the pandemic context, the COMET-G study (22) aimed at investigating levels of depression, changes in anxiety, distress, suicidal ideation, and spreading of conspiracy theories in relation with a number of personal and interpersonal variables. Some national level findings have already been reported (21, 23, 42–45) along with the comprehensive report of the international study (22).

The specific target of this national level investigation was to examine the individual and contextual factors associated with suicidality in the Romanian adult population in the context of the COVID-19 pandemic related lockdown, which provoked major societal turmoil.

The main objective was to investigate the association of suicidality with individual proximal disturbances (such as anxiety and depression) and a history of life-threatening events. We also hypothesized the following secondary aims to be scrutinized: (a) contextual factors such as adherence to conspiracy theories propagated through the classical media and the Internet would play a significant role in suicidality; in addition, traditional cultural factors such as religiosity would also influence the individual pattern of reality reading and subsequent suicidal ideation; (b) socio-demographic factors (such as age and level of education) would play a role in suicidality.

**Figure 1:**
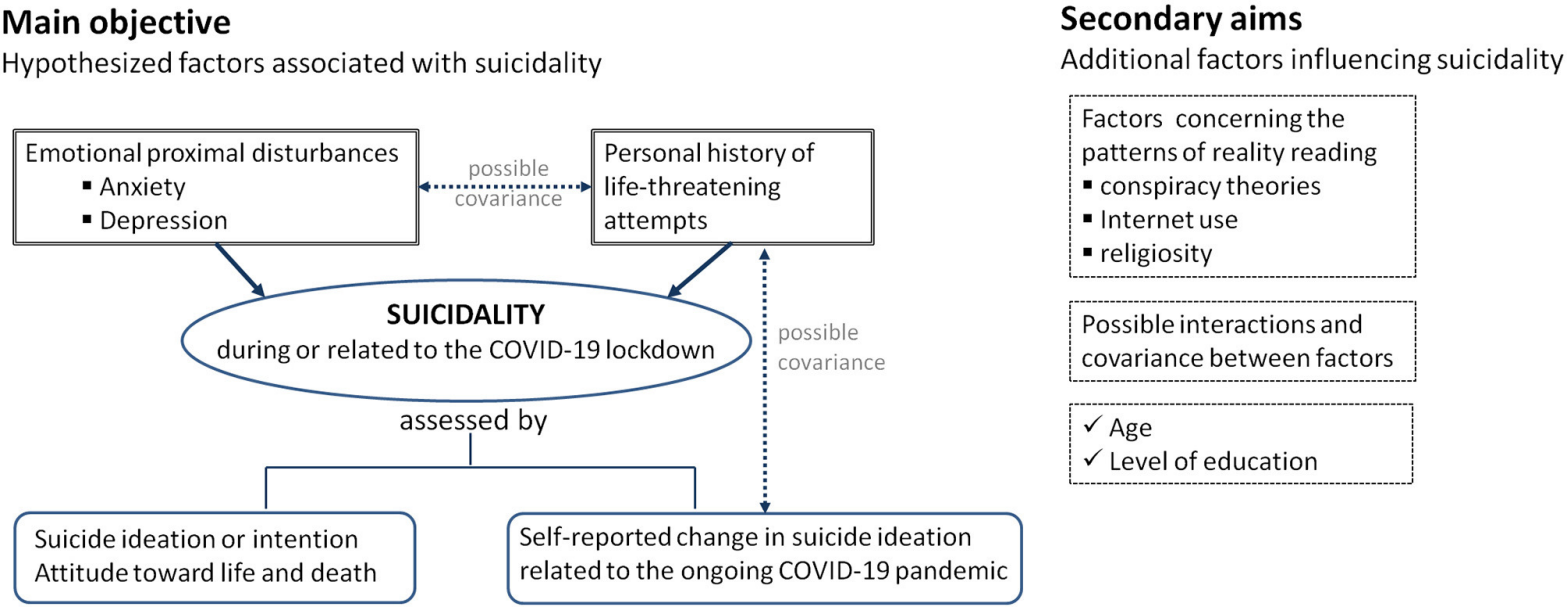
Main objective and secondary aims of this national level analysis of data collected on Romanian adult participants in the cross-sectional international COMET-G study.

Figure 1 illustrates the main objective and the secondary aims of this analysis. The conceptual framework included: suicidality, emotional disturbances, life threatening attempts and reality reading patterns. Suicidality refers to the “*risk of suicide, usually indicated by suicidal ideation or intent, especially as evident in the presence of a well-elaborated suicidal plan”* (46). Emotional disturbances comprise of three theoretical dimensions: emotional disturbances, emotion intensity/regulation disturbances, and emotion disconnections (47). Emotion intensity/regulation disturbances were mostly captured in the COMET-G study. Life threatening attempts encompassed the suicide attempts and the history of self-harm. Reality reading patterns would arise from the philosophical debate over the nature of conscious experience (48). The notion of indirect realism was extended to the reality perception in regard to the arising conspiracy theories, Internet use, and change in religiosity during the unfolding pandemic.

## Materials and Methods

### Participants and Procedures

The study followed the cross-sectional COMET-G study protocol (22). The anonymous questionnaires (available in the Supplementary Material 1) gathered demographic data, general health data, previous psychiatric history, current symptoms of anxiety, depression and suicidality, and data regarding the changes caused by the lockdown in sleep patterns, sexual life, family relationships, finances, eating behavior, physical exercising, and religiousness/spirituality. Beliefs regarding the COVID-19 outbreak, perceived efficacy of the lockdown measures, and conspiracy theories were also investigated.

The international questionnaire was translated into Romanian according to established standards (49). Independent translation and back translation were conducted by two Romanian- English speaking authors. Following the Delphi technique, a panel of professionals agreed upon the final version that was deployed.

Retrospective data were collected online from 1 June to 23 December, 2020 (total lockdown had been instituted in Romania from 16 March until 15 May, 2020). Participants were instructed to give answers referring to their state and mindset during the total lockdown. No identification information was collected. Participants were able to access the survey and complete their responses only after reading and acknowledging the information regarding the study (i.e., the cover story): aim of the research, organizations involved and their contact information, and planned use of collected data. This acknowledgment served as the on-line form of informed consent. Announcement and advertisements were placed on social media, and distributed *via* e-mail and other instant messaging Apps.

Ethical approval (no. 194/ 4 June, 2020) was issued by the Ethics Committee of the “Pius Brinzeu” County Emergency Clinical Hospital, in Timisoara, Romania.

### Instruments for Data Collection, Measures

Symptoms of anxiety were evaluated with State-Trait Anxiety Inventory (STAI), the S-Anxiety scale (STAI-Y1) (50). The STAI consists of 20 items that evaluate the respondent's current feelings on a 4-point Likert type scale. It is often employed for general and clinical populations (51) and had been used in Romania (52, 53).

Depression was evaluated with the Center for Epidemiological Studies-Depression Scale (CES-D), a popular and widely used instrument, based on self-reporting (54–56). It consists of 20 items that cover affective, psychological, and somatic symptoms (57), and had also been applied in Romanian population (56, 58–60).

Suicidality was evaluated with the Risk Assessment Suicidality Scale (RASS) (61), a self-assessment instrument. The last two RASS items were separately analyzed: RASS_11, “*Have you ever hurt yourself in any way deliberately, during your whole life so far?*”; RASS_12, “*Have you ever attempted suicide, during your whole life so far?*”. Each statement employed a 4-point Likert-type scale: 1 = not at all, 2 = a little bit, 3 = much, 4 = very much. An additional RASS-related item was included: “SI (Suicidal ideation) change,” “*How much has your tendency to think about death and/or suicide changed, compared to before the outbreak of COVID-19?*”. This instrument used a 5-point range: 2 = Very much increased, 1 = Increased a bit, 0 = Neither increased, nor decreased, −1 = Decreased a bit, −2 = Very much decreased. RASS had not been previously adapted for the Romanian population. Therefore, a confirmatory factor analysis (CFA) was conducted to verify its validity, based on the originally reported factors: “fear,” “intention,” and “life” (61).

Three additional Likert-type scales (designed for this study and included in the Supplementary Material 1) measured the extent of Internet use, belief in conspiracy theories, and the individual's religiosity. These scales underwent only face analysis prior to their deployment. CFA was conducted for the variables of Internet use and beliefs in conspiracy theories, which have been taken together as contributors to the patterns of reality reading.

### Definition of Latent Variables Based on the Manifest Exogenous Variables

Suicidality (*S*) was inferred from the total score of the first 10 items of RASS (*RASS tot*) and from the change in suicidal ideation (*SI change*). Observable emotional disturbances (*ED*) were measured by the total score for STAI-Y1 (*STAI tot*) and total score for CES-D (*CES tot*). Life threatening attempts (*LTA*) was a latent variable based on the items of RASS_11 (*RASS 11 self-harm*) and RASS_12 (*RASS 12 suicide*). Reality reading patterns (*RRP*) were inferred from the change in individual's religiosity or spirituality inquiries (*Relig increase*), belief in conspiracy theories (*Consp theories*), and *Internet use*.

### Data Analysis

#### Descriptive and Exploratory Statistics

Scale scores were treated as rank variables and described by the median (Inter Quartile Range). Descriptive statistics included the observed frequency counts (percent) for categorical variables or particular scales' selected items of interest. Normality of numerical variables was tested with the Shapiro-Wilk statistical test; these variables were described by the sample's mean and standard deviation (SD) when normally distributed, or by the sample's median (Inter Quartile Range–IQR) accordingly. The actual reliability of scale measurements was assessed based on the Cronbach's alpha: values >0.8 were considered to indicate good internal consistency, but scales with very few items were not discarded solely based on this coefficient. Cronbach's alpha actual values were reported for each scale with more than one item. The Harman's single factor method was applied to examine the amount of common method variance affecting the multi-item scales which had not undergone previous validation, other than face validation during the development stage. Harman's single factor method indicates possibly problematic common method bias (62). Separate application of the CFA marker technique to quantify the actual common method variance was unsuitable for scales taken in isolation, with possible additional issues related to the post-hoc choice of the marker (63). Non-parametric Spearman correlation approach was used to explore the covariances between various scales' scores employed in this study.

All reported probability values were two-tailed. A 0.05 level of significance was set, and highly significant values were also marked. Data were analyzed with the statistical software IBM SPSS v. 20.0 (Armonk, New York, USA) and the software packages R v. 4.0.5 (https://cran.r-project.org/).

#### Structural Equation Modeling and Confirmatory Factor Analysis

Based on the study specific target, structural equation modeling (SEM) was employed to investigate the structural connections between latent variables underlying the actual scores measured in the collected data. SEM was the method of choice for this analysis for its mathematical and statistical characteristics, i.e., a combination of model's structural features defined by equations, followed by their estimation across the available data based on the matrix algebra and generalized linear models. SEM is commonly used in the fields of social and psychological sciences for identifying hypothesized latent variables, which cannot be directly observed and measured. It also allows a simultaneous statistical estimation procedure, rather than separately estimating each part of a model, an approach which is believed to increase the overall accuracy (64).

We started with a nucleus model based on the main objective and its associated research hypotheses regarding the individual proximal disturbances (i.e., anxiety and depression) combined with a history of life-threatening attempts which would increase the suicidality related to the lockdown, thus including the endogenous latent variables of *ED, LTA*, and *S*. This model comprised previously validated scales as exogenous variables. In the following step, based on the secondary aims, we added the additional latent variable *RRP* in the model, which included the one-item change in individual's religiosity or spirituality inquiries, and the two multi-item scales for belief in conspiracy theories and Internet use (all three with only face validation). Furthermore, to this extended model we added two additional variables describing socio-demographic individual characteristics as potential independent predictors in the regression with *S* as an outcome. This approach yielded three SEM models, reflecting the results with reference to the main objective and the two secondary aims, respectively.

For all observed variables included in the models, the min-max rescaling was applied in order to preserve the shape of the original distributions and to retain the importance of outliers. The features would range as [0, 1] for all observed variables except for the change in suicidal thoughts, which was rescaled in the range [−1, 1] such that “no change” would correspond to a nil score. For model fitting, the maximum likelihood (ML) with robust estimators was used, with adjustments for non-normality of some variables (64, 65). The non-linear box-constrained optimization using PORT routines (NLMIB) was employed as the optimization method. When defining the SEM models, we placed the focus on the theoretical basis and meaningfulness of the variables' inter-relations. Nevertheless, the models were compared regarding their fit statistics and the Akaike information criterion (AIC). The Vuong's closeness test based on likelihood ratio was applied for determining the statistical significance of the change in AIC values.

The CFA and SEM models' goodness of fit indices and their corresponding [cut-off] values were: model Chi-square test and the resulting *p*-value, [< 0.05]; Comparative Fit Index (CFI), [> 0.90]; Root Mean Square Error of Approximation (RMSEA), [<0.08 for a good fit, and up to 0.1 for marginal fit]; Standardized Root Mean Square Residual (SRMR), [<0.08].

The levels of statistical confidence and significance were 0.95 and 0.05, respectively, except for the RMSEA fit index, for which the confidence was explicitly specified to be 0.90. All reported probability values were two-tailed. We conducted the analysis with the statistical packages R v. 4.0.5 (including “*lavaan*” v. 0.6-9, “*semPlot*” v. 1.1.2, and “*nonnest2*” v. 2020-07-05).

## Results

### Confirmatory Factor Analysis and Harman's Test for the Scales at Their First Deployment in Romanian Population

**Figure 2:**
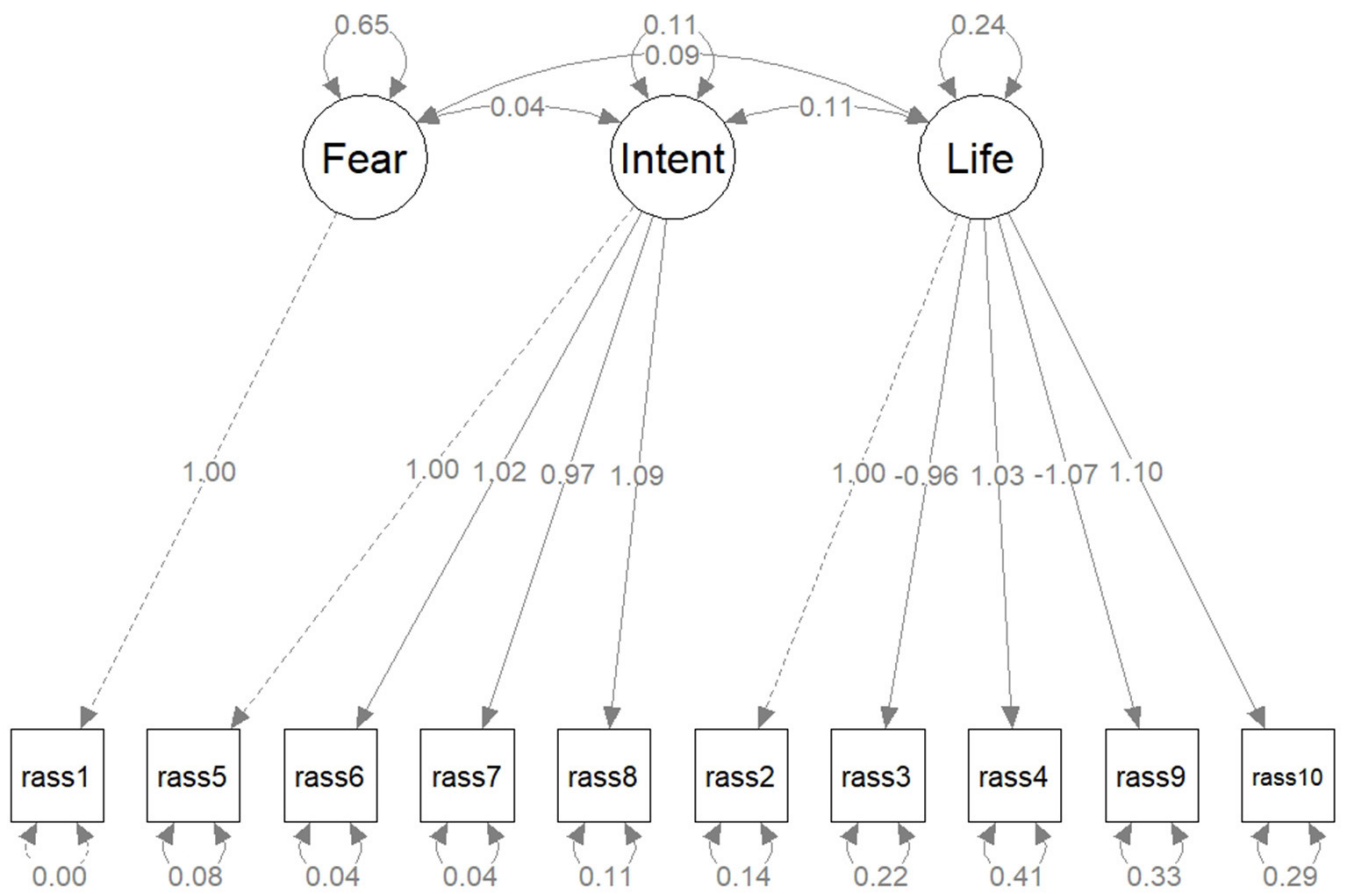
The path diagram for the confirmatory factor analysis for the first 10 items of the RASS scale, based on the three factors originally identified: fear, intention, and life. Latent variables are drawn in circles and manifest variables are drawn in squares. The edge labels indicate the parameter estimates.

CFA was conducted for the first 10 items of the RASS scale based on the three factors originally identified: fear, intention, and life. Results are presented in Table 1. All indices proved a good fit, except for the RMSEA, which was marginal. Figure 2 illustrates the CFA path diagram and factors' loadings, confirming the balanced contribution of all items to the overall score, with reversed effect for items #3 and #9. The actual RASS scale measurements were confirmed as consistent with scale's hypothesized construct.

**Table 1:** Confirmatory factor analysis (CFA) for the first 10 items of the RASS scale based on the three factors originally identified (fear, intention, and life) and the newly developed scales for belief in conspiracy theories (7 items) and Internet use (3 items).

**CFA model for the RASS scale**			
*Fear = ~ RASS_1* *Intention = ~ RASS_5 + RASS_6 + RASS_7 + RASS_8* *Life = ~ RASS_2 + RASS_3 + RASS_4 + RASS_9 + RASS_10*			
**Fit indices**			
**Chi-square test**	**CFI**	**RMSEA**	**SRMR**
417.374 (df = 33) *p* <0.001	0.941	0.090 90% CI (0.082; 0.098)	0.047
**CFA model for the variables of the beliefs in conspiracy theories and the Internet use**			
*Consp = ~ X.81_J1_ConspTheo_1 + X.82_J2_ConspTheo_2 + X.83_J3_ConspTheo_3 + X.84_J4_ConspTheo_4 + X.85_J5_ConspTheo_5 + X.86_J6_ConspTheo_6 + X.87_J7_ConspTheo_7* *Internet = ~ X.88_K1_Internet_1 + X.89_K2_Internet_2 + X.90_K3_Internet_3* *Consp ~~ Internet*			
**Fit indices**			
**Chi-square test**	**CFI**	**RMSEA**	**SRMR**
3992.427 (df = 45) *p* <0.001	0.910	0.085 90% CI (0.077; 0.093)	0.055

*Items are coded according to the COMET-G protocol as they are presented in the Supplementary Material 1*.

For the belief in conspiracy theories and Internet use, the Harman's single factor method resulted in 47.84 and 48.21%, respectively, of variance explained by one factor in exploratory factor analysis. These results on forced one factor model (namely less than 50% each) supported the further inclusion of the two scales in a SEM model. Table 1 also includes the CFA results for these two scales. Similarly to the 10-item RASS scale, the fit indices were good, except for the RMSEA, which was marginal.

### Descriptive Analysis of Socio-Demographic Characteristics, Self-Reported Health Data and Mental Disturbances of the Respondents

One thousand, four hundred and forty-six (*N* = 1,446) adults responded to the anonymous questionnaire: 1,142 were females (aged 46.83 ± 14.16 years), 292 were males (aged 47.64 ± 14.36 years), and 12 self-identified as “non-binary” (aged 51.58 ± 15.45 years). Details of the respondents' socio-demographic information, and data regarding education and employment are presented in Tables 2A,B, respectively. Additional self-reported health related data were included in Supplementary Material 2.

**Table 2A:** Socio-demographic characteristics of the respondents.

**Variable**	**Total** ***N*** **= 1,446**	**Female** ***N*** **= 1,142**	**Male** ***N*** **= 292**	**Non-binary** ***N*** **= 12**
**Age (years)**				
mean ± std.dev.	47.03 ± 14.21	46.83 ± 14.165	47.64 ± 14.36	51.58 ± 15.45
(min–max)	(19–84)	(19–84)	(19–80)	(21–82)
**Residence**	*n* (% of N)	*n* (% of N)	*n* (% of N)	*n* (% of N)
Rural area–village	262 (18.1%)	218 (19.1%)	40 (13.7%)	4 (33.3%)
Town (<20.000 inhabitants)	189 (13.1%)	148 (13%)	39 (13.4%)	2 (16.7%)
Town (20.000–100.000 inhabitants)	347 (24%)	278 (24.3%)	68 (23.3%)	1 (8.3%)
City (100.000–1 million population)	471 (32.6%)	357 (31.3%)	113 (38.7%)	1 (8.3%)
City > 1 million population	70 (4.8%)	62 (5.4%)	8 (2.7%)	–
Capital city	107 (7.4%)	79 (6.9%)	24 (8.2%)	4 (33.3%)
**Marital status**	*n* (% of N)	*n* (% of N)	*n* (% of N)	*n* (% of N)
Single	224 (15.5%)	176 (15.4%)	46 (15.8%)	2 (16.7%)
Married (or in a civil partnership)	860 (59.5%)	657 (57.5%)	200 (68.5%)	3 (25%)
Divorced (or estranged)	98 (6.8%)	84 (7.4%)	14 (4.8%)	–
Live with someone without an official relationship	155 (10.7%)	128 (11.2%)	25 (8.6%)	2 (16.7%)
Widower	84 (5.8%)	80 (7%)	2 (0.7%)	2 (16.7%)
Other	25 (1.7%)	17 (1.5%)	5 (1.7%)	3 (25%)
**Household people**	*n* (% of N)	*n* (% of N)	*n* (% of N)	*n* (% of N)
1	195 (13.5%)	166 (14.5%)	26 (8.9%)	3 (25%)
2	522 (36.1%)	401 (35.1%)	119 (40.8%)	2 (16.7%)
3	373 (25.8%)	294 (25.7%)	76 (26%)	3 (25%)
4	235 (16.3%)	187 (16.4%)	46 (15.8%)	2 (16.7%)
5	121 (8.4%)	94 (8.2%)	25 (8.6%)	2 (16.7%)
**Children**	n (% of N)	n (% of N)	n (% of N)	n (% of N)
0	416 (28.8%)	326 (28.5%)	88 (30.1%)	2 (16.7%)
1	494 (34.2%)	403 (35.3%)	87 (29.8%)	4 (33.3%)
2	414 (28.6%)	323 (28.3%)	88 (30.1%)	3 (25%)
3	79 (5.5%)	59 (5.2%)	19 (6.5%)	1 (8.3%)
4	43 (3%)	31 (2.7%)	10 (3.4%)	2 (16.7%)

**Table 2B:** The respondents' education and employment data.

**Variable**	**Total** ***N*** **= 1,446**	**Female** ***N*** **= 1,142**	**Male** ***N*** **= 292**	**Non-binary** ***N*** **= 12**
**Education**	*n* (% of N)	*n* (% of N)	*n* (% of N)	*n* (% of N)
Elementary school	46 (3.2%)	35 (3.1%)	6 (2.1%)	5 (41.7%)
High school (9–12 yrs)	366 (25.3%)	265 (23.2%)	99 (33.9%)	2 (16.7%)
Bachelor degree	652 (45.1%)	521 (45.6%)	128 (43.8%)	3 (25%)
University	89 (6.2%)	78 (6.8%)	11 (3.8%)	–
MA (MSc) degree	254 (17.6%)	216 (18.9%)	37 (12.7%)	1 (8.3%)
PhD	39 (2.7%)	27 (2.4%)	11 (3.8%)	1 (8.3%)
**Employment**	*n* (% of N)	*n* (% of N)	*n* (% of N)	*n* (% of N)
Civil servant	463 (32%)	398 (34.9%)	63 (21.6%)	2 (16.7%)
Private clerk	314 (21.7%)	222 (19.4%)	88 (30.1%)	4 (33.3%)
Self-employed/freelancer	91 (6.3%)	67 (5.9%)	23 (7.9%)	1 (8.3%)
Retired	284 (19.6%)	215 (18.8%)	66 (22.6%)	3 (25%)
Unemployed	16 (1.1%)	10 (0.9%)	6 (2.1%)	–
Housekeeper	56 (3.9%)	55 (4.8%)	1 (0.3%)	–
Disability pension	21 (1.5%)	17 (1.5%)	4 (1.4%)	–
Allowance for health reasons	5 (0.3%)	4 (0.4%)	–	1 (8.3%)
University or college student	123 (8.5%)	100 (8.8%)	23 (7.9%)	–
Other	73 (5%)	54 (4.7%)	18 (6.2%)	1 (8.3%)
**Health sector**	*n* (% of N)	*n* (% of N)	*n* (% of N)	*n* (% of N)
No	1,099 (76%)	837 (73.3%)	252 (86.3%)	10 (83.3%)
Doctor	67 (4.6%)	58 (5.1%)	9 (3.1%)	–
Nurse	201 (13.9%)	182 (15.9%)	19 (6.5%)	–
Other healthcare profession	55 (3.8%)	47 (4.1%)	6 (2.1%)	2 (16.7%)
Administrative staff in hospital	9 (0.6%)	6 (0.5%)	3 (1%)	–
Other hospital staff	15 (1%)	12 (1.1%)	4 (1%)	–

Table 3 presents the descriptive statistics for the scales' scoring totals, and the corresponding values of Cronbach's alpha for individual scales or RASS sub-scales (i.e., as they resulted from the CFA). Lower values of internal consistency can be noted for the three-item “Internet use” (alpha = 0.456). For the three individual RASS items (O11, O12, and O13), the median (IQR) statistics were all nil. Table 4 shows the distribution of the scores for these individual items and also includes the scoring distribution for the change in religious/spiritual inquiries, where the actual spread over the whole range is apparent.

**Table 3:** Descriptive statistics and internal consistency of scale scorings for STAI, CES, RASS, belief in conspiracy theories, and internet use.

**Scale Median (IQR)**	**Total** ***N*** **= 1,466**	**Female** ***N*** **= 1,142**	**Male** ***N*** **= 292**	**Non-binary** ***N*** **= 12**
**STAI total**
Sum (F1, F2,..., F20)
Cronbach's alpha = 0.922 (20 items)
**STAI total**	48 (39–55)	49 (40–56)	43 (36–52)	43.50 (29–53)
**CES total**
Sum (G1, G2,..., G20)
Cronbach's alpha = 0.927 (20 items)
**CES total**	12 (6–24)	13 (6–24)	10 (4–19)	9.5 (2.5–26)
**RASS total**
Sum (O1, O2,..., O10)
RASS fear = {O1}
RASS intention = {O5, O6, O7, O8}, Cronbach's alpha = 0.894 (4 items)
RASS life = {O2, O3, O4, O9, O10}, Cronbach's alpha = 0.825 (5 items)
**RASS total**	6 (6–8)	6 (6–8)	6 (6–7)	6 (6–7)
**Consp total**
Sum (J1, J2,..., J7)
Cronbach's alpha = 0.677 (7 items)
**Consp total**	8 (4–12)	8 (4–12)	8 (4–12)	14 (10.5–18)
**Internet total**
sum (K1, K2, K3)
Cronbach's alpha = 0.456 (3 items)
**Internet total**	4 (3–6)	5 (3–6)	4 (3–6)	3.5 (1.5–7)

*Items are coded according to the COMET-G protocol as they are presented in the Supplementary Material 1*.

**Table 4:** The scorings' distributions for the individual items on suicidality change, personal history of self-harm, and increase in religious/spiritual inquiries.

	**RASS N (%)**	**Total** ***N*** **= 1,466**	**Female** ***N*** **= 1,142**	**Male** ***N*** **= 292**	**Non-binary** ***N*** **= 12**
**Subjective changes in suicidality** **(O11_Suicidality change)**		*n* (% of N)	*n* (% of N)	*n* (% of N)	*n* (% of N)
	−2	90 (6.2%)	67 (5.9%)	23 (7.9%)	–
	−1	20 (1.4%)	15 (1.3%)	4 (1.4%)	1 (8.3%)
	0	1,171 (81%)	918 (80.4%)	244 (83.6%)	9 (75%)
	1	105 (7.3%)	93 (8.1%)	12 (4.1%)	–
	2	60 (4.1%)	49 (4.3%)	9 (3.1%)	2 (16.7%)
**History of self-harm (O12_RASS_11)**		*n* (% of N)	*n* (% of N)	*n* (% of N)	*n* (% of N)
	0	1,299 (89.8%)	1,020 (89.3%)	268 (91.8%)	11 (91.7%)
	1	77 (5.3%)	65 (5.7%)	12 (4.1%)	–
	2	41 (2.8%)	32 (2.8%)	9 (3.1%)	–
	3	29 (2%)	25 (2.2%)	3 (1%)	1 (8.3%)
**History of suicide attempts** **(O13_RASS_12)**		*n* (% of N)	*n* (% of N)	*n* (% of N)	*n* (% of N)
	0	1,339 (92.6%)	1,055 (92.4%)	275 (94.2%)	9 (75%)
	1	79 (5.5%)	65 (5.7%)	12 (4.1%)	2 (16.7%)
	2	23 (1.6%)	19 (1.7%)	4 (1.4%)	–
	3	5 (0.3%)	3 (0.3%)	1 (0.3%)	1 (8.3%)
**Changes in religiousness/spirituality (P1_RelSpir)**		*n* (% of N)	*n* (% of N)	*n* (% of N)	*n* (% of N)
	0	672 (46.5%)	482 (42.2%)	185 (63.4%)	5 (41.7%)
	1	419 (29%)	352 (30.8%)	65 (22.3%)	2 (16.7%)
	2	203 (14%)	171 (15%)	31 (10.6%)	1 (8.3%)
	3	152 (10.5%)	137 (12%)	11 (3.8%)	4 (33.3%)

*Items are coded according to the COMET-G protocol as they are presented in the Supplementary Material 1*.

Correlation between the scale scorings is presented in Table 5. The strong correlation between STAI and CES-D is noteworthy, although all scorings (except for the belief in conspiracy theories) were significantly two-by-two correlated. The belief in conspiracy theories showed a very weak or no relation to anxiety, depression, suicidality and Internet use.

**Table 5:** Associations between the scales total scorings on anxiety, depression, suicidality, conspiracy beliefs and Internet use.

		**STAI total**	**CES-D total**	**RASS total**	**Conspiracy total**	**Internet total**
**STAI total**	R	1.000	0.776**	0.358**	0.085**	0.334**
	p	.	<0.001	<0.001	0.001	<0.001
	N	1,446	1,446	1,446	1,446	1,446
**CES-D total**	R	0.776**	1.000	0.355**	0.119**	0.370**
	p	<0.001	.	<0.001	0.000	<0.001
	N	1,446	1,446	1,446	1,446	1,446
**RASS total**	R	0.358**	0.355**	1.000	−0.019	0.211**
	p	<0.001	<0.001	.	0.477	<0.001
	N	1,446	1,446	1,446	1,446	1,446
**Conspiracy total**	R	0.085**	0.119**	−0.019	1.000	0.177**
	p	0.001	<0.001	0.477	.	<0.001
	N	1,446	1,446	1,446	1,446	1,446
**Internet total**	R	0.334**	0.370**	0.211**	0.177**	1.000
	p	<0.001	<0.001	<0.001	<0.001	.
	N	1,446	1,446	1,446	1,446	1,446

Statistical significance **p <0.01.

*N, number of observations; p, statistical significance; R, Spearman coefficient of correlation (non-parametric). Statistically significant R values over 0.3 are in bold*.

### Structural Equation Models

Table 6 shows the SEM models along with their statistical fit indices. We started with the nucleus Model 1, with reference to the main objective, which included three latent variables (*ED, LTA*, and *S*) and a regression (*ED* and *LTA* as predictors for S). In Model 2, we added an additional latent variable (*RRP*), which was included in the regression, as well. Model 3 kept the same latent variables, and also incorporated age and education as independent predictors in the regression. For all three models, we also investigated meaningful covariance. According to the Vuong's statistical test and the AIC, each model gave successively better description of the variables inter-relations, when compared to the previous one. For all three models, the fits indices reflected good reliability.

**Table 6:** The structural equation modeling of the multivariable relationships between the mental health indicators, beliefs and life changes.

**SEM models**	**Fit indices**			
	**Chi-square test**	**CFI**	**RMSEA**	**SRMR**
**Model 1** *ED = ~ STAI total + CES total* *Suicidality = ~ RASS total + SI change* *LTA = ~ RASS_11 + RASS_12* *SI change~~ RASS_11 + RASS_12* *Suicidality ~ ED + LTA*	11.272 (df = 4) *p*= 0.024	0.997	0.035 90% CI (0.012; 0.061)	0.010
**Model 2** *ED = ~ STAI total + CES total* *Suicidality = ~ RASS total + SI change* *LTA = ~ RASS_11 + RASS_12* *RRP = ~ Religion and spirituality + Conspiracy theories + Internet use* *SI change~~ RASS_11 + RASS_12* *Internet use ~~ Conspiracy theories* *Suicidality ~ ED + LTA + RRP*	130.038 (df = 18) *p* <0.001	0.959	0.066 90% CI (0.055; 0.076)	0.040
Vuong's test: z = 6.244; *p* <0.001 (in favor of Model 2, compared to Model 1) 95% CI of AIC difference (−812.220; −414.567)	
**Model 3** *ED = ~ STAI total + CES total* *Suicidality = ~ RASS total + SI change* *LTA = ~ RASS_11 + RASS_12* *RRP = ~ Religion and spirituality + Conspiracy theories + Internet use* *SI change~~ RASS_11 + RASS_12* *Internet use ~~ Conspiracy theories* *Suicidality ~ ED + LTA + RRP + Age + Education*	305.938 (df = 34) *p* <0.001	0.906	0.074 90% CI (0.067; 0.082)	0.058
Vuong's test: z = 2.227, *p* = 0.013 (in favor of Model 3, compared to Model 2) 95% CI of AIC difference (−35.591; 1.475)				

Table 7 presents the parameters for the SEM model 3 in detail. *ED* and previous *LTA* were significant predictors for *S*, while the *RRP* were not. In addition, the participants' age was a significant predictor (with negative regression coefficient), but the level of education was not. It is important to note the significant covariance between each of the three latent variables considered as predictors in the regression, namely *ED, LTA*, and *RRP*; there was a significant negative covariance between the previous *LTA* (RASS_11 and RASS_12 items) and the reported change in SI during the COVID-19 pandemic lockdown. There was no covariance between Internet use and beliefs in conspiracy theories.

**Table 7:** The parameters of SEM Model 3 examining the relationships between anxiety, depression, life-threatening attempts, suicidality, religion/spirituality, conspiracy theories, Internet use scorings, age, and education.

**Model 3 parameters**		**Estimate (Std. err.)**	**z-value**	***p*** **-value**
Latent variables:
ED = ~	STAI total	1		
	CES total	1.209 (0.044)	27.356	<0.001**
Suicidality = ~	RASS total	1		
	SI change	1.568 (0.261)	6.001	<0.001**
LTA = ~	RASS_11	1		
	RASS_12	0.753 (0.103)	7.341	<0.001**
RRP = ~	Religion and spirituality	1		
	Conspiracy theories	0.664 (0.125)	5.309	<0.001**
	Internet use	1.228 (0.227)	5.413	<0.001**
Regression:
Suicidality ~	ED	0.168 (0.027)	6.251	<0.001**
	LTA	0.316 (0.063)	5.047	<0.001**
	RRP	0.036 (0.052)	0.678	0.497
	Age	−0.049 (0.013)	−3.865	<0.001**
	Education	0.014 (0.012)	1.142	0.253
Covariances:
SI change~~	RASS_11	−0.007 (0.003)	−2.401	0.016*
	RASS_12	−0.007 (0.002)	−3.207	0.001**
Internet use ~~	Conspiracy theories	−0.00019 (0.002)	−0.088	0.930
ED ~~	LTA	0.007 (0.001)	6.423	<0.001**
	RRP	0.010 (0.002)	5.696	<0.001**
LTA ~~	RRP	0.002 (0.001)	3.457	0.001**

Statistical significance *p <0.05; **p < 0.01.

**Figure 3:**
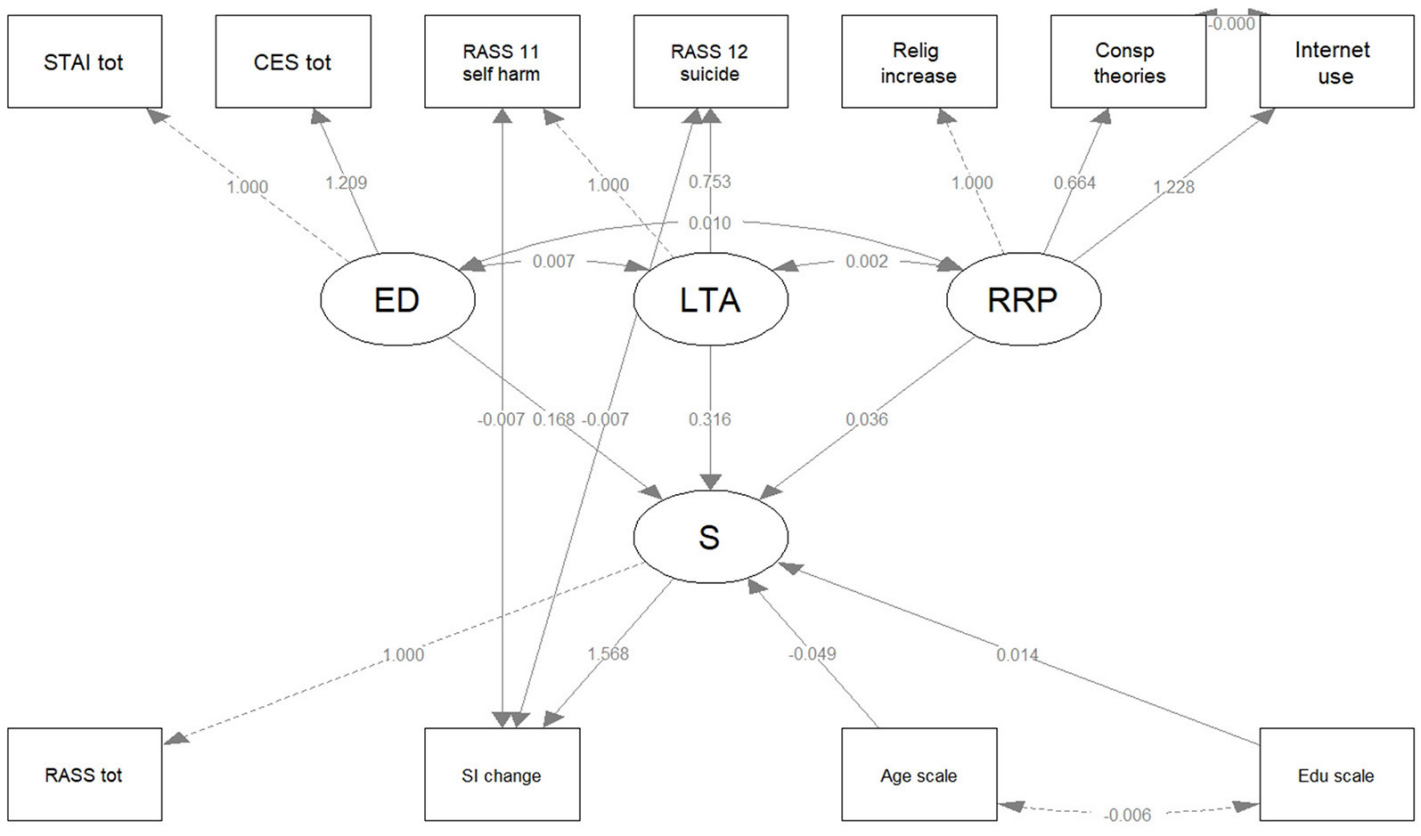
The path diagram for the SEM Model 3. Latent variables are drawn in circles and manifest variables are drawn in squares. The edge labels indicate the parameter estimates. CES tot, Center for Epidemiologic Studies Depression Scale (CES-D), 20-item total; ED, Emotional Disturbances; LTA, Life Threatening Attempts; RASS 11 self-harm, 4-point score of RASS 11; RASS 12 suicide, 4-point score of RASS 12; RASS tot, Risk Assessment Suicidality Scale (RASS), 10-item total; S, Suicidality; SI change, Suicidal Ideation change; STAI tot, State-Trait Anxiety Inventory (STAI), 20-item total.

Figure 3 shows the path diagrams for the SEM model 3. The latent variables are drawn in circles; the manifest variables are drawn in squares.

The parameters of the SEM model 1 and model 2, and their corresponding path diagrams are presented in Supplementary Material 3.

## Discussion

The present study on a lot of 1,446 Romanian adult participants in the international cross-sectional COMET-G study included persons aged between 19 and 84 years, with a mean of 47.03 years. More than 50% of the 1,446 respondents self-declared an increased level of religiosity and spiritual inquiries during the COVID-19 lockdown in the pandemic outbreak. Eighty-one percent self-reported no suicidality change, but more than 11% reported increased suicidal ideation during the lockdown. More than 10% of the 1,446 respondents admitted having a history of self-harm and more than 7% reported previous suicide attempts. In the structural models of suicidality, emotional disturbances and previous life-threatening attempts acted as significant predictors, while the patterns of reality reading were not. Young age was also a significant predictor for suicidality. The construct of suicidality was based on the RASS total scoring and the change in suicidal ideation. Of the two, the change in suicidal ideation played a more consistent role. For emotional disturbances, both STAI-Y1 total scoring (anxiety) and CES-D total scoring (depression) contributed in a similar way. Previous life-threatening attempts were observed in terms of two items of the RASS scale regarding the self-harm and previous suicide attempts, both contributing to life-threatening attempts in almost equal terms. The patterns of reality reading encompassed the adherence to conspiracy theories, Internet use, and change in spirituality inquiries. The Internet use had the highest estimate and the conspiracy beliefs the lowest, although both had high statistical significance for reality reading patterns. Although the SEM models 2 and 3 which included them were significantly better compared to the nucleus model, their contribution to the suicidality proved insignificant. They might only indirectly contribute through their significant covariance with the emotional disturbances.

We compared the socio-demographic characteristics for our responders with the officially reported data on the general population of Romania (66–69): median age of 43.2 years, rural residence of 43.6% in the general population (compared to 18.1% among the respondents), 61.1% married (59.5% in our data set), 4.84% unemployed (1.1% in our data set), 51.4 % females (78.9% in our data set). Summing up, compared to the general population of Romania, the respondents in the present study were of similar age, higher urban representation, similar marital status, higher employment status, and higher female representation. In particular, the dissimilarities in females' proportion and unemployment rates could have an impact on the models' validity, due to their previously reported effect on suicidality. Despite these concerns, the rate of Internet users in Romania is high and 12 million people use social media in Romania, a country with 19.18 million citizens (66, 70), therefore we confidently chose the on-line means to promote the COMET-G study. Although the respondents' sample was not totally representative for the general population, the number of respondents was high, compared to other countries cited in the COMET-G project (22).

Suicidality and self-harm history are widely acknowledged as substantial predictors for suicidal risk (71, 72), but the evidence is largely based on data from high-income countries (18). Data regarding the Romanian population is particularly scarce. In our models, previous life-threatening attempts proved to be significant predictors for suicidality. An intriguing finding was that both factors – suicide attempts and self-harm history – were negatively correlated with the change in suicidal ideation, albeit the correlation was weak (but statistically significant).

There are published reports of decreased suicidal ideation in association with the pandemic outbreak in Europe and the United States (18, 73, 74). Suicidal ideation might decrease when people are confronted with immediate potentially existential dangers, such as the risk of illness and the sense of incertitude during the COVID-19 pandemic. There are established observations of this phenomenon in other situations of immediate threats, like the First World War or terrorist attacks (75, 76). Depression was long seen as a suicide-related factor, but the effect of anxiety has not been separately investigated until recently (77, 78). The context of the COVID-19 pandemic might have also mediated a more direct connection between the increased anxiety and suicidality, as it unmasked and developed multiple anxiety-generating factors such as the fear of contamination, general insecurity, fear for the loved ones' health, and subject's overexposure on the media. What seems especially intriguing in this specific context is that the suicide rates were stable and suicidality was reported as decreasing; in a context when the general rates of the risk factors for suicidality (such as depression, anxiety, contextual and social vulnerabilities) increased in most of the reports on the COVID-19 pandemic and the contribution of these factors is well-established in the literature, alerts for constant vigilance regarding the suicidal dynamic were issued (18).

Age is usually inversely correlated with suicidal risk (4), and our SEM model 3 also put younger people at a higher risk. Studies on Romanian population showed a significant rise in suicide for young people and the elderly, even before the pandemic crisis (79). Lower education levels is typically seen as a general risk factor for suicidality, but it loses influence when adding other dominant factors, such as preexisting mental health issues, ancestry information, and demographic factors (7, 80, 81). In our SEM model 3, the level of education was not a significant predictor for suicidality. On the other hand, this lack of education significance in our model might be due to the disequilibrium in the level of education among the respondents: fewer than 30% of them did not graduate a form of post-high- school education. As these data were somewhat incongruous and unforeseen in the pandemic context, further investigations for long-term consequences in stationary societal circumstances are necessary, accompanied by national policies aimed at this public health issue.

There is a consensus that most people are quite resilient in face of negative changes or potentially traumatic events (82–84). Onset of societal or economic instability (for example, a recession) may have unstructured effects on suicide rates (85, 86). Nevertheless, vulnerability factors (such as previous mental health issues, suicide attempts, a history of self-harm, male sex, age, unemployment and belonging to disadvantaged social groups) may influence the life-long mental health risks, and indeed play an important role in the suicidality dynamics (77, 87–89). Religion generally plays a protective role regarding suicide (90), while religious turmoil is associated with a greater suicidal risk (91), albeit moderated by specific cultural differences (92). Responses to the COMET-G questionnaire showed that self-reported change in spiritual inquiries may have acted as a signal that previously successful coping mechanisms might have been exhausted, and the increased religiosity could thus be viewed as an attempt to regain emotional balance. Conspiracist ideation is also grounded on psychological mechanisms (93) and tends to increase during times of crises (94). Moreover, current media misinformation seems to generate a specific dynamic that exacerbates and promotes conspiracy thinking (95). These mechanisms were initially hypothesized to also work in the Romanian adult population, but in our structural models the conspiracy beliefs did not correlate with the degree of Internet use and did not demonstrate a significant influence on suicidality. This might be explained by the methodology we used that raises issues of consistency and common method bias for the variables related to the reality reading patterns. Both findings need further investigation and additional channels for proliferation of conspiracy theories should be considered. Specific scales, thoroughly validated, are also needed for clarification.

The pandemic context calls for consideration of new factors related to suicide. This implies that measures already established as being protective might require reconsideration and adjustment in the near future. For example, anxiety disorder and anxiety related distress emerged as a significant suicidality factor in the present study, thus needing deeper scrutiny in further research. We put forward a particular need for consolidation of the presently proposed structural models of suicidality.

Worldwide, several different studies have proposed vulnerability models for mental health issues (21, 42, 96) while suicide-related studies of the Romanian population have found it as atypical and therefore faced difficulties in applying models from other Eastern European Countries (97). Romania presents with a set of challenges regarding the medical system and with several cultural and socio-economic particularities, some of which are widely acknowledged as associating with higher suicidal risk. However, the national suicide rates have slightly declined over the past years, recently falling below the annual global age-standardized suicide rate (14). Precaution was recommended in regard to the pandemic consequences (98), but recent results showed a degree of psychological stability during the lockdown in Romanian population, and studies have indicated no change in suicide rates for some regions of Romania (99). However, little overall data is available, so the present results may bring valuable contribution toward moving forward with the novel understanding of suicidality.

### Limitations

The main limitation of this investigation is that the proposed SEM models of suicidality were generated based on anonymous self-reported data, which were retrospectively collected in regard to the COVID-19 lockdown, within a limited time window and based on natural self-selection of respondents. Its cross-sectional design with no previous baseline and no follow-up prevented us from obtaining the risk estimates. Nevertheless, the SEM procedure in data analysis allowed the combination of the structural features with a general linear model for regression, and increased the overall accuracy and subsequent reliability of the findings.

The common method bias (CMB) implied by the cross-sectional design and the one time single-administration questionnaire (with its associated actual effect of the common method variance, CMV) is a major concern that cannot be overlooked. On the other hand, appropriate procedural measures were taken and carefully observed to limit the shared variance and control the method biases: different scales (such as those corresponding to predictors and criterion constructs) were included in non-adjacent sections, separated by questions collecting factual data (e.g., about diet or physical exercising); the scales included both positively and negatively (i.e., reverse) worded items; the wording was kept clear, concise and accurate; at the beginning of the questionnaire, respondents were provided comprehensive information on the COMET-G study and were assured of the anonymity; different scale formats were alternated, such as 4-point and 5-point Likert-type scales, or even dichotomy items.

In addition to these preventative measures, we explored the CMB possible impact on the performance of the measuring instruments and subsequent results by the post-hoc statistical techniques. Moreover, the approach with three SEM models (the nucleus including only previously validated scales and widely acknowledged constructs) and the stability of these nuclei regression coefficients' estimates (i.e., proximal predictors) across the three models proved the robustness of the results: significant and balanced interrelationship between the nuclei constructs (i.e., emotional disturbances and previous life-threatening attempts on the one hand, and suicidality on the other hand). We acknowledge that CMV, as a systematic error variance, could have a confounding influence on empirical results and produce potentially misleading conclusions, but this issue was improbable in our case. In our data set, there was a weak and insignificant relationship between the beliefs in conspiracy theories and the use of Internet – an actual CMB issue should have resulted in a stronger relationship.

Additional concerns might arise from employing scales not previously applied in the Romanian population, such as the RASS scale. Not only was the translation endorsed following a Delphi technique, but the CFA did support the original structure of the scale. The scales comprised in the construct of reality reading patterns proved to be less consistent and this issue should be addressed more carefully in the future. In addition, certain recall bias was possibly included in the answers.

Furthermore, this cross-sectional survey of self-reported perceived changes selectively recruited respondents who habitually navigate on the Internet, so the response rate was difficult to estimate, and acceptable rate was also problematic to anticipate or gauge. Moreover, this approach in questionnaire distribution led to a certain bias toward the population favorable toward on-line instruments, and this might have affected their appreciation toward the information and communication technology, thus the inconsistency on the scale of Internet use (it might have been too simplistic for many respondents).

The lack of follow-up imposes limits on the proposed models' external validity. An additional caveat regarding the validity originates in the pronounced gender disequilibrium among the respondents, which presumably reflect the degree of Internet engagement, but would affect the models' cross-gender validity for the rate of suicide completion is greater among males.

## Conclusion

Suicidality has specific particularities for each country, region, or cultural context and environment, and the results we report bring evidence toward improving the insights into the Romanian population. Suicidality also has a context related inner dynamic, but affective disturbances, history of suicide attempts and self-harm remain the main factors related to suicide risk even in the special context of the COVID-19 pandemic in Romania. The final suicidality construct we developed also related to the COVID-19 lockdown, for this was specifically mentioned in the cover story of the questionnaire.

Our results confirmed anxiety and depression as significant proximal predictors in suicidality. In spite of every effort we made to answer the secondary aims of this study, the issue of quantifying the reality reading patterns' influence on suicidality remains open and must yet be further investigated.

Because suicide has a disastrous impact on the immediate family, it brings trans-generational mental health vulnerability. This investigation contributes to a better understanding of suicidality in a specific context, and may thus serve as a guide for assessing risks and identifying effective interventions.

## Data Availability Statement

The raw data supporting the conclusions of this article will be made available by the authors, without undue reservation.

## Ethics Statement

The studies involving human participants were reviewed and approved by Pius Brinzeu County Emergency Clinical Hospital, in Timisoara, Romania. The patients/participants provided their written informed consent to participate in this study.

## Author Contributions

All authors listed have made a substantial, direct, and intellectual contribution to the work and approved it for publication.

## Conflict of Interest

The authors declare that the research was conducted in the absence of any commercial or financial relationships that could be construed as a potential conflict of interest.

## Publisher's Note

All claims expressed in this article are solely those of the authors and do not necessarily represent those of their affiliated organizations, or those of the publisher, the editors and the reviewers. Any product that may be evaluated in this article, or claim that may be made by its manufacturer, is not guaranteed or endorsed by the publisher.

## Abbreviations and Nodes in Sem Models

- *Age scale*, age on a 6-group scale of adult age;
- *CES tot*, Center for Epidemiologic Studies Depression Scale (CES-D), 20-item total;
- *Consp theories*, 7-item total of belief in conspiracy theories;
- *ED, Emotional Disturbances*, latent variable describing self-reported fear, anxiety, depression;
- *Edu scale*, level of education on a 5-item scale;
- *Internet use*, 3-item total of Internet use;
- *LTA, Life Threatening Attempts*, latent variable describing suicide-related personal history;
- *RASS 11 self-harm*, 4-point score of RASS 11 (“Have you ever harmed yourself in any way deliberately, during your whole life so far?”);
- *RASS 12 suicide*, 4-point score of RASS 12 (“Have you ever attempted suicide, during your whole life so far?”);
- *RASS tot*, Risk Assessment Suicidality Scale (RASS), 10-item total;
- *Relig increase*, 4-point score of increase of religious/spiritual inquiries (“Over the last two-three weeks, my religious/spiritual inquiries have been increased.”);
- *RRP, Reality Reading Patterns*, latent variable describing personal vulnerabilities related to social and spiritual issues;
- *S, Suicidality*, latent variable describing suicidality;
- *SI change, Suicidal Ideation change*, 5-point score of change in suicidal ideation;
- *STAI tot*, State-Trait Anxiety Inventory (STAI), 20-item total.
