# Supplementary material for: Suicidality Related to the COVID-19 Lockdown in Romania: Structural Equation Modeling
Source: Front Psychiatry. 2022 May 17;13:818712. doi: 10.3389/fpsyt.2022.818712 (PMC9152167; doi:10.3389/fpsyt.2022.818712)
Supplement: Supplementary file 1 [file Data_Sheet_1.docx]

Supplementary Material 1

COMET-G study questionnaire

**Α. GENERAL DATA**

**A1. Sex**

- • Male
- • Female
- • Other/I do not wish to define

**A2. Date of Birth written with 4 digits (e.g. 1982)**

Please enter your date of birth in the form of 4 digits (e.g. 1981, 1968, 1993 etc.)

**A3. Place of residence during this period**

- • Capital City
- • City > 1 million population
- • City (100.000 – 1 million population)
- • Town (20.000 – 100.000 inhabitants)
- • Town (<20.000 inhabitants)
- • Rural area - Village

**A4. Marital Status**

- • Single
- • Married (or in a civil partnership)
- • Divorced (or estranged)
- • Live with someone without an official relationship
- • Widower
- • Other

**A5. How many people reside in the house you are staying in during this period (including yourself)?**

- • 1 (I live alone)
- • 2 people
- • 3 people
- • 4 people
- • 5 or more people

**A6. How many children do you have (regardless of whether they live in the same house with you or not)?**

- • 0 (I do not have any children)
- • 1 child
- • 2 children
- • 3 children
- • 4 or more children

**A7. Education**

- • Elementary school or less (no more than 9 years of education)
- • High school degree or equivalent (no more than 12 years of education)
- • Bachelor’s degree
- • Master’s degree
- • Doctorate (PhD)

**A8. Employment**

- • Working at the Public Sector
- • Salaried employee at the Private Sector
- • Self-employed/Freelancer
- • Retired
- • Unemployed
- • Housekeeping
- • Retirement for health reasons
- • Allowance for health reasons
- • Not working by choice (e.g. living via corporate earnings etc.)
- • University or College Student
- • Other

**A9. Employment in the Health Sector**

- • I do not work in the Heath Sector
- • Doctor
- • Nurse
- • Other healthcare profession with direct contacts with areas of clinical work
- • Administrative employee at a hospital
- • Other hospital staff

**A10. Do you continue to exercise your profession during the period of the lockdown?**

- • Yes
- • No

**B. GENERAL STATE OF HEALTH AND COVID-19**

**B1. In general, your health over the last month can be described as:**

- • Excellent
- • Very good
- • Good
- • Moderate
- • Bad

**B2. Do you suffer from any chronic medical condition (for example: diabetes mellitus, hypertension, asthma, etc.)?**

- • Yes
- • No

**B3. If yes, please define your chronic medical condition:**

**B4. Are you a close relative or caretaker of a person that belongs to a vulnerable group?**

- • Yes
- • No

**B5. In the past, have you had any mental health problem serious enough to make you seek professional health, psychotherapy or medication treatment?**

- • No
- • Anxiety
- • Depression
- • Psychosis
- • Bipolar Disorder
- • Other

**B6. Are you currently under any kind of treatment for your mental state? (please check all of those that are true)**

- • No
- • Psychotherapy
- • Antipsychotics
- • Antidepressants
- • Tranquilizers**/**benzodiazepines (lexotanil, xanax, tavor, etc.)

**C. THOUGHTS ABOUT COVID-19**

**C1. Are you afraid that you will contract the coronavirus;**

- • Never
- • A little
- • Moderately
- • Much
- • Very Much

**C2. Do you believe that the precautions work effectively or that if you are about to contract the disease, you will contract it anyway?** 7

- • Precautions work effectively.
- • Precautions cannot protect you.

**C3. Does the possibility that a member of your family could contract the coronavirus and die because of it makes you frightened?**

- • Never
- • A little
- • Moderately
- • Much
- • Very Much

**C4. Are you afraid that in case you contract the coronavirus, some people will step away from your life and behave to you in a different way later?**

- • Never
- • A little
- • Moderately
- • Much
- • Very Much

**D. THOUGHTS ABOUT THE MEASURES TAKEN (precautions, lockdown, etc.)**

**D1. According to your opinion, the amount of time that you spend outside of your house for reasons not regarding your work during this period is:**

- • Minimum
- • Less than humanly necessary
- • Moderate**/**reasonable
- • Enough
- • A lot
- • Excessive

**D2. Are you currently locked up in the house?**

- • Completely
- • To a high degree
- • Partially
- • Not at all

**D3. According to the instructions given by WHO, it is necessary for some precautions to be taken in order to prevent the spread of the virus. Do you believe that you take adequate precautions?**

- • Not at all
- • A little bit
- • Moderately
- • Much
- • Very Much

**D4. Do you believe that that you have received sufficient information about the necessity of the measures taken?**

**•** • Not at all

• • A little bit

• • Moderately

• • Much

• • Very Much

**E. FAMILY**

**E1. Do you feel the need to communicate with other members of your family during this period?**

- • Much less
- • Less
- • Same
- • More
- • Much more

**E2. Do you want to receive emotional support from other members of your family during this period?**

- • Much less
- • Less
- • Same
- • More
- • Much more

**E3. Are there any conflicts with the rest of your family members during this period?**

- • Much less
- • Less
- • Same
- • More
- • Much more

**E4. Has the overall quality of relationships with the other members of your family changed compared to the one before the quarantine, due to COVID – 19?**

- • Much worse
- • Worse
- • It has not changed
- • A little bit better
- • Much better

**E5. Do you manage to maintain a basic daily routine (waking up in the morning, regular meals and sleeping hours, activities) both yourself (if you live alone) or as a family?** 9

- • Not at all
- • Somehow, but not always
- • Generally, yes
- • Clearly follow (or adhere to) a routine

**E6. If you have children, how difficult is it to manage their daily life and behavior?**

- • Much more difficult than before
- • Somehow more difficult but not always
- • Same as always
- • Somehow easier but not always
- • Much easier than before

**E7. How are your finances as a result of the outbreak?**

- • Much more difficult than before
- • Somehow more difficult
- • Same as always
- • Somehow easier
- • Much easier than before

**F. ANXIETY**

State-Trait Anxiety inventory

STAI-Y1 questions: F1-F20

**F21. How much has your emotional state changed in relation to the appearance of anxiety and insecurity compared to before the COVID-19 epidemic?**

- • It got a lot worse
- • It got a little worse
- • Neither better nor worse
- • It’s a little improved
- • It has improved a lot

**G. SADNESS & DEPRESSION/MELANCHOLY**

Center for Epidemiological Studies-Depression Scale (CES-D): G1-G20

**G1. I was bothered by things that usually don’t bother me.**

- • Rarely or none of the time (less than 1 day)
- • Some of a little of the time (1-2 days)
- • Occasionally or a moderate amount of time (3-4 days)
- • Most or all of the time (5-7 days)

**G2. I did not feel like eating; my appetite was poor.**

- • Rarely or none of the time (less than 1 day)
- • Some of a little of the time (1-2 days)
- • Occasionally or a moderate amount of time (3-4 days)
- • Most or all of the time (5-7 days)

**G3. I felt that I could not shake off the blues even with help from my family or friends.**

- • Rarely or none of the time (less than 1 day)
- • Some of a little of the time (1-2 days)
- • Occasionally or a moderate amount of time (3-4 days)
- • Most or all of the time (5-7 days)

**G4. I felt I was just as good as other people.**

- • Rarely or none of the time (less than 1 day)
- • Some of a little of the time (1-2 days)
- • Occasionally or a moderate amount of time (3-4 days)
- • Most or all of the time (5-7 days)

**G5. I had trouble keeping my mind on what I was doing.**

- • Rarely or none of the time (less than 1 day)
- • Some of a little of the time (1-2 days)
- • Occasionally or a moderate amount of time (3-4 days)
- • Most or all of the time (5-7 days)

**G6. I felt depressed.**

- • Rarely or none of the time (less than 1 day)
- • Some of a little of the time (1-2 days)
- • Occasionally or a moderate amount of time (3-4 days)
- • Most or all of the time (5-7 days)

**G7. I felt that everything I did was an effort.**

- • Rarely or none of the time (less than 1 day)
- • Some of a little of the time (1-2 days)
- • Occasionally or a moderate amount of time (3-4 days)
- • Most or all of the time (5-7 days)

**G8. I felt full of hope about the future.**

- • Rarely or none of the time (less than 1 day)
- • Some of a little of the time (1-2 days)
- • Occasionally or a moderate amount of time (3-4 days)
- • Most or all of the time (5-7 days)

**G9. I thought my life had been a failure.**

- • Rarely or none of the time (less than 1 day)
- • Some of a little of the time (1-2 days)
- • Occasionally or a moderate amount of time (3-4 days)
- • Most or all of the time (5-7 days)

**G10. I felt fearful.**

- • Rarely or none of the time (less than 1 day)
- • Some of a little of the time (1-2 days)
- • Occasionally or a moderate amount of time (3-4 days)
- • Most or all of the time (5-7 days)

**G11. My sleep was restless.**

- • Rarely or none of the time (less than 1 day)
- • Some of a little of the time (1-2 days)
- • Occasionally or a moderate amount of time (3-4 days)
- • Most or all of the time (5-7 days)

**G12. I was happy.**

- • Rarely or none of the time (less than 1 day)
- • Some of a little of the time (1-2 days)
- • Occasionally or a moderate amount of time (3-4 days)
- • Most or all of the time (5-7 days)

**G13. I talked less than usual.**

- • Rarely or none of the time (less than 1 day)
- • Some of a little of the time (1-2 days)
- • Occasionally or a moderate amount of time (3-4 days)
- • Most or all of the time (5-7 days)

**G14. I felt lonely.**

- • Rarely or none of the time (less than 1 day)
- • Some of a little of the time (1-2 days)
- • Occasionally or a moderate amount of time (3-4 days)
- • Most or all of the time (5-7 days)

**G15. People were unfriendly.**

- • Rarely or none of the time (less than 1 day)
- • Some of a little of the time (1-2 days)
- • Occasionally or a moderate amount of time (3-4 days)
- • Most or all of the time (5-7 days)

**G16. I enjoyed life.**

- • Rarely or none of the time (less than 1 day)
- • Some of a little of the time (1-2 days)
- • Occasionally or a moderate amount of time (3-4 days)
- • Most or all of the time (5-7 days)

**G17. I had crying spells.**

- • Rarely or none of the time (less than 1 day)
- • Some of a little of the time (1-2 days)
- • Occasionally or a moderate amount of time (3-4 days)
- • Most or all of the time (5-7 days)

**G18. I felt sad.**

- • Rarely or none of the time (less than 1 day)
- • Some of a little of the time (1-2 days)
- • Occasionally or a moderate amount of time (3-4 days)
- • Most or all of the time (5-7 days)

**G19. I felt that people disliked me.**

- • Rarely or none of the time (less than 1 day)
- • Some of a little of the time (1-2 days)
- • Occasionally or a moderate amount of time (3-4 days)
- • Most or all of the time (5-7 days)

**G20. I could not get “going”.**

- • Rarely or none of the time (less than 1 day)
- • Some of a little of the time (1-2 days)
- • Occasionally or a moderate amount of time (3-4 days)
- • Most or all of the time (5-7 days)

**G21. How much has your emotional state related to the experience of joy or melancholy changed in comparison to before the COVID-19 epidemic?**

- • It got a lot worse
- • It got a little worse
- • Neither better nor worse
- • Improved a bit
- • It has improved a lot

**H. PHYSICAL ACTIVITY**

**H1. Does exercise help you at the prevention of anxiety?**

- • Not at all
- • A little bit
- • Moderately
- • Much
- • Very much

**H2. Do you consider that exercise is important during this pandemic?**

- • Not at all
- • A little bit
- • Moderately
- • Much
- • Very much

**H4. How much has your physical activity been affected by this epidemic of COVID-19?**

- • It decreased much
- • It decreased a little
- • Neither decreased, nor increased
- • It increased a little
- • It increased much

**H3. Do you have increased the frequency and intensity of your physical workout during this pandemic and lockdown?**

• • Not at all

• • A little bit

• • Moderately

• • Much

• • Very much

**I. NUTRITION**

**I1. During the days of the lockdown did you notice the need to eat larger amounts of food or eat more often?**

- • I eat much less than I used to
- • I eat bit less than I used to
- • Neither more nor less
- • I eat a bit more than I used to
- • I eat much more than I used to

**I2. Please mark the answer that best represents you during the period of the lockdown:**

- • I eat in a healthier way.
- • My eating habits and preferences have not changed.
- • I eat in a more unhealthy way.

**I3. Please mark the answer that best represents you during the period of the lockdown:**

- • My body weight has significantly decreased (more than 2-3 kilos).
- • My body weight has slightly decreased (less than 2 kilos lost).
- • My body weight is stable.
- • My body weight has slightly increased (less than 2 kilos put).
- • My body weight has significantly increased (more than 2-3 kilos).

**J. OPINIONS ABOUT THE ORIGIN OF THE EPIDEMIC**

**J1. Do you believe that the COVID-19 vaccine was ready even before the virus broke out and they conceal it from us for the benefit of pharmaceutical companies?**

- • I don’t believe it at all
- • A little bit
- • Maybe
- • Much
- • Very much

**J2. Do you believe that COVID-19 was created in a laboratory to be used as a biochemical weapon for the extermination of the human population?**

- • I don’t believe it at all
- • A little bit
- • Maybe
- • Much
- • Very much

**J3. Do you believe that COVID-19 is the result of 5G technology antenna?**

- • I don’t believe it at all
- • A little bit
- • Maybe
- • Much
- • Very much

**J4. Do you believe that COVID-19 appeared accidentally from human contact with animals and it was something that generally happens and was generally expected?**

- • I don’t believe it at all
- • A little bit
- • Maybe
- • Much
- • Very much

**J5. Do you believe that COVID-19 has much lower mortality rate but there is misinformation and terror-inducing propaganda?**

- • I don’t believe it at all
- • A little bit
- • Maybe
- • Much
- • Very much

**J6. Do you believe that COVID-19 is a creation of the world’s powerful leaders to create a global economic crisis?**

- • I don’t believe it at all
- • A little bit
- • Maybe
- • Much
- • Very much

**J7. Do you believe that COVID-19 is a sign of divine power to destroy our planet?**

- • I don’t believe it at all
- • A little bit
- • Maybe
- • Much
- • Very much

**K. INTERNET**

**K1. The information and use of the internet worry me about the issue regarding the COVID-19:**

- • Not at all
- • A little
- • Moderately
- • Much
- • Very much

**K2. Generally, most of the internet sources regarding information about COVID-19 are misinforming/misleading:**

- • Not at all
- • A little
- • Moderately
- • Much
- • Very much

**K3. Due to the conditions, the internet takes up more of my time than usual:**

- • Not at all
- • A little more
- • Moderately more
- • Much more
- • Too much

**K4. How much do you use the social media while in isolation at home?**

- • More than before
- • The same as before
- • Less than before

**K5. Have you acquired internet-related habits that you did not have before (for example: created a facebook account, engaging in cybersex or gambling)?**

- • Yes
- • No

**L. SLEEP DURING THIS PERIOD**

**L1. The quality of my sleep has changed recently. It is:**

- • Much worse
- • A little bit worse
- • The same (neither worse nor better)
- • A little better
- • Much better

**L2. I tend to stay up late and sleep for many hours during the day.**

- • Almost never
- • Rarely
- • Sometimes
- • Often
- • Almost always

**L3. I take sleeping pills to help me sleep at night.**

- • Almost never
- • Rarely
- • Some times
- • Often
- • Almost always

**L4. I am having dreams in which I feel trapped, over the last 3 weeks.**

- • Almost never
- • Rarely
- • Sometimes
- • Often
- • Almost always

**M. SMOKING, ALCOHOL AND SUBSTANCES USE**

**M1. Smoking before the epidemic:**

- • I didn't smoke
- • I was smoking

**M2. Alcohol use before the epidemic:**

- • I did not drink much
- • I drank a lot (more than one drink or its equivalent every day)

**M3. Use of illegal substances before the epidemic (e.g. hashish):**

- • I did not use it
- • Occasionally and rather rarely
- • Often

**M4. During lockdown, you smoked compared to before:**

- • More than before
- • Same as before
- • Less than before

**M5. During lockdown, you drinked alcohol compared to before:**

- • More than before
- • Same as before
- • Less than before

**M6. While isolated at home, you used illegal substances compared to before:**

- • More than before
- • Same as before
- • Less than before

**N. SEXUALITY**

**N1. How would you characterize the frequency of your sexual intercourse?**

- • Clearly inadequate
- • Rather inadequate
- • Neutral
- • Rather satisfactory
- • Clearly satisfactory

**N2. Due to the new lockdown conditions, has your desire for sexual intercourse increased or decreased?**

- • Has decreased a lot
- • Has decreased slightly
- • Remains the same
- • Has increased slightly
- • Has increased a lot

**N3. How much pleasure and satisfaction do you get from your current sex life?**

- • Not at all
- • A little bit
- • Moderate
- • Much
- • Very much

**N4. Do you think sex helps you deal with your daily stress and anxiety?**

- • Not at all
- • A little bit
- • Moderate
- • Much
- • Very much

**O. THOUGHTS ABOUT DEATH**

Risk Assessment Suicidality Scale (RASS): O1 – O12

**O1** **_RASS_1. Are you afraid that you are going to die?**

• • Not at all

• • A little bit

• • Much

• • Very much

**O2** **_RASS_2. Do you ever think that it would be better if you were dead?**

- • Not at all
- • A little bit
- • Much
- • Very much

**O3** **_RASS_3. Do you think that it is a wonderful thing that you are alive?**

- • Not at all
- • A little bit
- • Much
- • Very much

**O4** **_RASS_4. Have you ever felt that it’s worth living?**

- • Not at all
- • A little bit
- • Much
- • Very much

**O5** **_RASS_5. Do you think of harming yourself physically?**

- • Not at all
- • A little bit
- • Much
- • Very much

**O6** **_RASS_6. Do you often think of committing suicide if you have the chance?**

- • Not at all
- • A little bit
- • Much
- • Very much

**O7** **_RASS_7. Do you make plans concerning the method to use in order to end your life?**

- • Not at all
- • A little bit
- • Much
- • Very much

**O8** **_RASS_8. I am thinking of committing suicide, but I won’t do it.**

- • Not at all
- • A little bit
- • Much
- • Very much

**O9** **_RASS_9. Do you enjoy your life?**

- • Not at all
- • A little bit
- • Much
- • Very much

**O10** **_RASS_10. Are you feeling tired from your life?**

- • Not at all
- • A little bit
- • Much
- • Very much

**O11. How much has your tendency to think about death and/or suicide changed, compared to before the outbreak of COVID-19?**

- • Very much increased
- • Increased a bit
- • Neither increased, nor decreased
- • Decreased a bit
- • Very much decreased

**O12** **_RASS_11. Have you ever hurt yourself in any way deliberately, during your whole life so far?**

- • Never
- • Once
- • 2-3 times
- • Many times

**O13** **_RASS_12. Have you ever attempted suicide, during your whole life so far?**

- • Never
- • Once
- • 2-3 times
- • Many times

**P. SPIRITUALITY AND PSYCHOLOGICAL REFECTIONS**

**P1. Over the last 2-3 weeks, my religious/spiritual inquiries have been increased.**

- Not at all.
- A little bit
- Much
- Very Much
