# Supplementary material for: Suicidality Related to the COVID-19 Lockdown in Romania: Structural Equation Modeling
Source: Front Psychiatry. 2022 May 17;13:818712. doi: 10.3389/fpsyt.2022.818712 (PMC9152167; doi:10.3389/fpsyt.2022.818712)
Supplement: Supplementary file 2 [file Data_Sheet_2.docx]

Supplementary Material 2

# The respondents' health-related data

**Supplementary Table 2.1**. The respondents' general health data

| **Variable** | **Total**  N=1446 | **Female**  N=1142 | **Male**  N=292 | **Non-binary**  N=12 |
| --- | --- | --- | --- | --- |
| **Lockdown Exercise** | n (% of N) | n (% of Nvalid) | n (% of Nvalid) | n (% of Nvalid) |
| 0 (no) | 220 (15.2%) | 178 (21.4%) | 40 (18.6%) | 2 (25%) |
| 1 (yes) | 844 (79.3%) | 663 (78.8%) | 175 (81.4%) | 6 (75%) |
| missing | 382 (26.4%) | ‒ | ‒ | ‒ |
| **Last Month Health Condition** | n (% of N) | n (% of N) | n (% of N) | n (% of N) |
| 0 (bad) | 64 (4.4%) | 57 (5%) | 7 (2.4%) | ‒ |
| 1 | 176 (12.2%) | 155 (13.6%) | 20 (6.8%) | 1 (8.3%) |
| 2 | 552 (38.2%) | 442 (38.7%) | 107 (36.6%) | 3 (25%) |
| 3 | 449 (31.1%) | 342 (29.9%) | 105 (36%) | 2 (16.7%) |
| 4 (excellent) | 205 (14.2%) | 146 (12.8%) | 53 (18.2%) | 6 (50%) |
| **Somatic Chronic Medical Condition** | n (% of N) | n (% of N) | n (% of N) | n (% of N) |
| 0 (no) | 795 (55%) | 632 (55.3%) | 158 (54.1%) | 6 (50%) |
| 1 (yes) | 651 (45%) | 511 (44.6%) | 134 (45.9%) | 6 (50%) |
| **Vulnerable Group** | n (% of N) | n (% of N) | n (% of N) | n (% of N) |
| 0 (no) | 841 (58.2%) | 640 (56%) | 190 (65.1%) | 11 (91.7%) |
| 1 (yes) | 605 (41.8%) | 502 (44%) | 102 (34.9%) | 1 (8.3%) |

**Supplementary Table 2.2**. The respondents' mental health data

| **Variable** | **Total**  N=1446 | **Female**  N=1142 | **Male**  N=292 | **Non-binary**  N=12 |
| --- | --- | --- | --- | --- |
| **Past Mental Health Problem** | n (% of N) | n (% of N) | n (% of N) | n (% of N) |
| No | 1148 (79.4%) | 897 (78.5%) | 243 (83.2%) | 8 (66.7%) |
| Anxiety | 98 (6.3%) | 84 (7.4%) | 14 (4.8%) | ‒ |
| Depression | 161 (11.1%) | 134 (11.7%) | 24 (8.2%) | 3 (25%) |
| Psychosis | 5 (0.3%) | 1 (0.1%) | 4 (1.4%) | ‒ |
| Bipolar Disorder | 12 (0.8%) | 7 (0.6%) | 4 (1.4%) | 1 (8.3%) |
| Other | 22 (1.5%) | 19 (1.7%) | 3 (1%) | ‒ |
| **Current Treatment Mental State** | n (% of N) | n (% of N) | n (% of N) | n (% of N) |
| No | 1293 (89.4%) | 1015 (88.9%) | 268 (91.8%) | 10 (83.3%) |
| Psychotherapy | 25 (1.7%) | 22 (1.9%) | 3 (1%) | ‒ |
| Antipsychotics | 10 (0.7%) | 4 (0.4%) | 6 (2.1%) | ‒ |
| Antidepressants | 75 (5.2%) | 64 (5.6%) | 10 (3.4%) | 1 (8.3%) |
| Tranquilizers/benzodiazepines (lexotanil, xanax, tavor, etc.) | 43 (3%) | 37 (3.2%) | 5 (1.7%) | 1 (8.3%) |
