# Supplementary material for: Suicidality Related to the COVID-19 Lockdown in Romania: Structural Equation Modeling
Source: Front Psychiatry. 2022 May 17;13:818712. doi: 10.3389/fpsyt.2022.818712 (PMC9152167; doi:10.3389/fpsyt.2022.818712)
Supplement: Supplementary file 3 [file Data_Sheet_3.docx]

Supplementary Material 3

# Results of Structural Equation Modeling Analysis: Model 1 and Model 2

**Supplementary Table 3.1**. The parameters of SEM Model 1 examining the relationships between anxiety, depression and suicidality scorings

| **Model 1 parameters** | | **Estimate (Std. err.)** | **z-value** | **p-value** |
| --- | --- | --- | --- | --- |
| Latent Variables: | | | | |
| ED =~ | STAI total | 1 |  |  |
|  | CES total | 1.279 (0.055) | 23.190 | <0.001** |
| Suicidality =~ | RASS total | 1 |  |  |
|  | SI change | 1.724 (0.227) | 6.225 | <0.001** |
| LTA =~ | RASS_11 | 1 |  |  |
|  | RASS_12 | 0.725 (0.100) | 7.213 | <0.001** |
| Regression: | | | | |
| Suicidality ~ | ED | 0.174 (0.021) | 8.301 | <0.001** |
|  | LTA | 0.323 (0.064) | 5.042 | <0.001** |
| Covariances: | | | | |
| .SI change~~ | .RASS_11 | -0.007 (0.003) | -2.527 | 0.011* |
|  | .RASS_12 | -0.007 (0.002) | -3.311 | 0.001** |
| ED ~~ | LTA | 0.007 (0.001) | 6.421 | <0.001** |
| **Note**: Statistical significance * p < 0.05; ** p < 0.01 | | | | |

**Supplementary Table 3.2**. The parameters of SEM Model 2 examining the relationships between anxiety, depression, suicidality, religion/spirituality, conspiracy theories and internet use scorings

| **Model 2 parameters** | | **Estimate (Std. err.)** | **z-value** | **p-value** |
| --- | --- | --- | --- | --- |
| Latent Variables: | | | | |
| ED =~ | STAI total | 1 |  |  |
|  | CES total | 1.216 (0.045) | 27.288 | <0.001** |
| Suicidality =~ | RASS total | 1 |  |  |
|  | SI change | 1.692 (0.275) | 6.145 | <0.001** |
| LTA =~ | RASS_11 | 1 |  |  |
|  | RASS_12 | 0.730 (0.101) | 7.239 | <0.001** |
| RRP =~ | Religion and spirituality | 1 |  |  |
|  | Conspiracy theories | 0.665 (0.126) | 5.296 | <0.001** |
|  | Internet use | 1.230 (0.228) | 5.388 | <0.001** |
| Regression: | | | | |
| Suicidality ~ | ED | 0.164 (0.027) | 6.019 | <0.001** |
|  | LTA | 0.322 (0.064) | 5.053 | <0.001** |
|  | RRP | 0.034 (0.052) | 0.652 | 0.514 |
| Covariances: | | | | |
| .SI change~~ | .RASS_11 | -0.007 (0.003) | -2.484 | 0.013* |
|  | .RASS_12 | -0.007 (0.002) | -3.298 | 0.001** |
| .Internet use ~~ | .Conspiracy theories | -0.0002 (0.002) | -0.094 | 0.925 |
| ED ~~ | LTA | 0.007 (0.001) | 6.454 | <0.001** |
|  | RRP | 0.010 (0.002) | 5.670 | <0.001** |
| LTA ~~ | RRP | 0.002 (0.001) | 3.458 | 0.001** |
| **Note:** Statistical significance * p < 0.05; ** p < 0.01 | | | | |


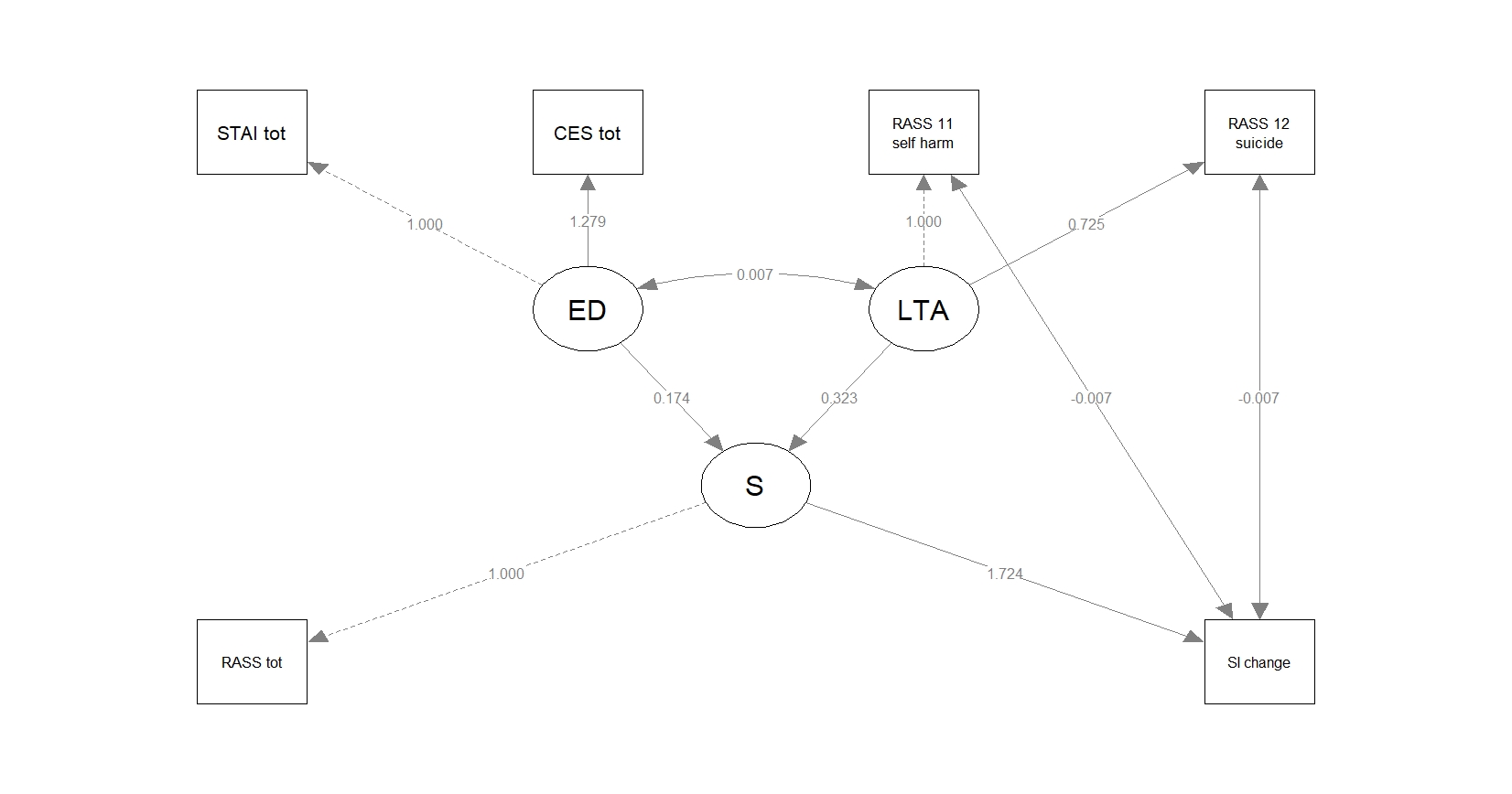


**Supplementary Figure 3.1.** The path diagram for the SEM Model 1. Latent variables are drawn in circles and manifest variables are drawn in squares. The edge labels indicate the parameter estimates.


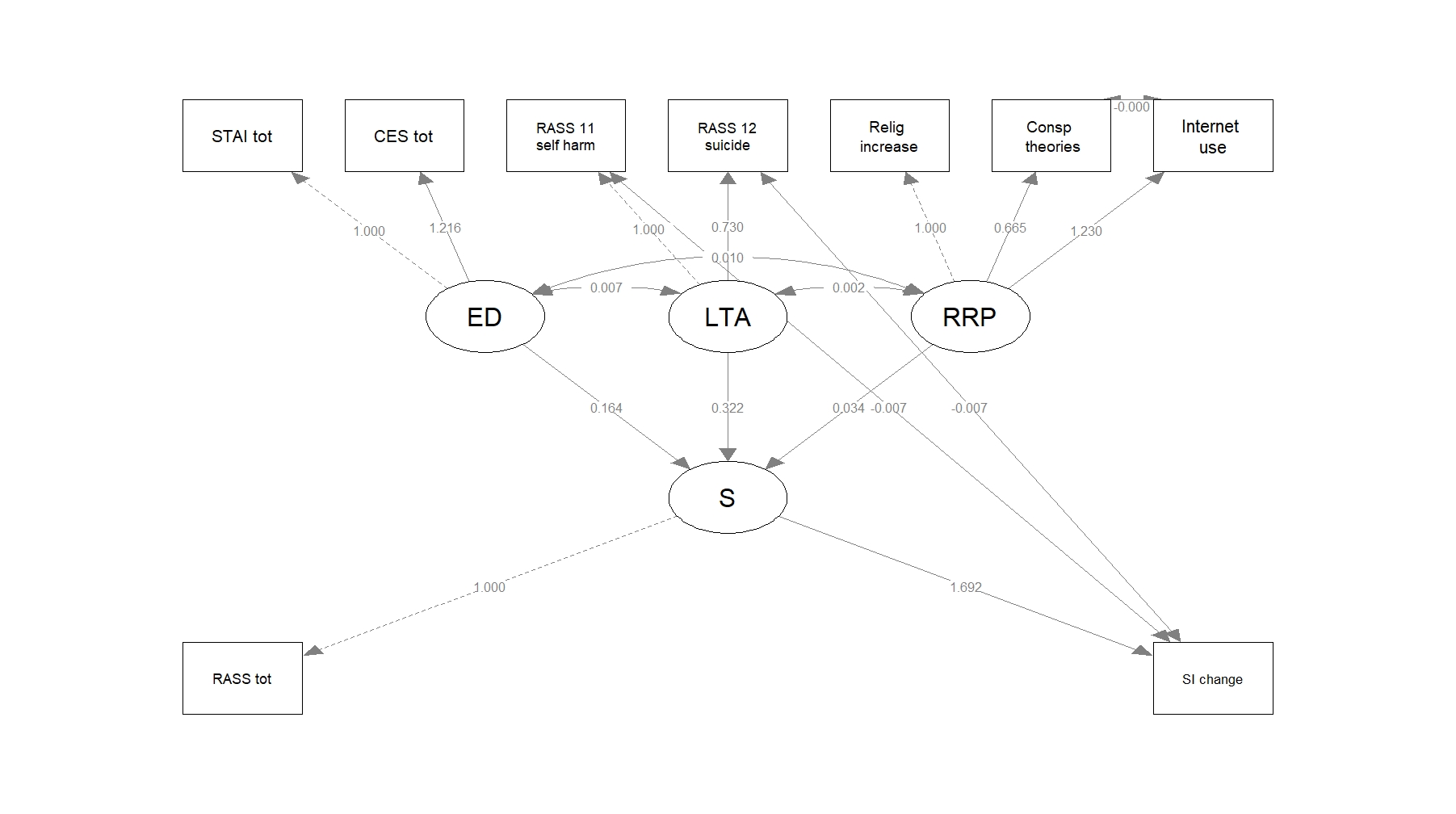


**Supplementary Figure 3.2.** The path diagram for the SEM Model 2. Latent variables are drawn in circles and manifest variables are drawn in squares. The edge labels indicate the parameter estimates.

**Abbreviations in the figures are**: **CES tot**, Center for Epidemiologic Studies Depression Scale (CES-D), 20-item total; **ED,** Emotional Disturbances; **LTA,** Life Threatening Attempts; **RASS 11 self-harm**, 4-point score of RASS 11; **RASS 12 suicide**, 4-point score of RASS 12; **RASS tot**, Risk Assessment Suicidality Scale (RASS), 10-item total; ***RRP****, Reality Reading Patterns;* **S,** Suicidality; **SI change,** Suicidal Ideation change; **STAI tot**, State-Trait Anxiety Inventory (STAI), 20-item total.
